# Aggression toward Familiar People, Strangers, and Conspecifics in Gonadectomized and Intact Dogs

**DOI:** 10.3389/fvets.2018.00018

**Published:** 2018-02-26

**Authors:** Parvene Farhoody, Indika Mallawaarachchi, Patrick M. Tarwater, James A. Serpell, Deborah L. Duffy, Chris Zink

**Affiliations:** ^1^Psychology Department, The Graduate Center of the City University of New York, New York, NY, United States; ^2^Department of Biomedical Sciences, Texas Tech University Health Sciences Center at El Paso, El Paso, TX, United States; ^3^Department of Biostatistics, University of Texas Health Science Center at Houston, Houston, TX, United States; ^4^Department of Clinical Studies, University of Pennsylvania, Philadelphia, PA, United States; ^5^Office of Institutional Research and Effectiveness, University of the Arts, Philadelphia, PA, United States; ^6^Zink Integrative Sports Medicine, Ellicott City, MD, United States

**Keywords:** gonadectomy, dog, neuter, spay, aggression, behavior

## Abstract

Gonadectomy is widely used to treat and prevent behavior problems including the aggressive behavior of dogs. The aim of this study was to determine whether aggressive behavior toward familiar people, strangers, or other dogs was significantly different in dogs gonadectomized at various ages vs. intact dogs using the Canine Behavioral Assessment Research Questionnaire (C-BARQ) with multivariate analysis. Of 15,370 initial surveys, those for dogs reported to have been gonadectomized at less than 6 weeks of age or to correct a behavior problem, and those with incomplete answers to questions regarding independent or dependent variables were excluded, leaving 13,795 for the analysis of aggressive behavior toward familiar people: 13,498 for aggressive behavior toward strangers and 13,237 for aggressive behavior toward dogs. Aggressive behavior was defined (a) using mean scores for all questions on the C-BARQ for aggressive behavior (range 0–4) and (b) comparing dogs with no aggressive behavior (all questions answered 0) to dogs with moderate or severe aggression (at least one score of 2, 3, or 4). Data for intact dogs were compared with those for dogs gonadectomized at 6 months or less, 7–12 months, 11–18 months, and >18 months. Neither gonadectomy nor age at gonadectomy showed an association with aggression toward familiar people or dogs. However, there was a low but significant increase in the odds of moderate or severe aggression toward strangers for all gonadectomized dogs compared with intact dogs, but this effect was driven entirely by data for dogs gonadectomized at 7–12 months of age, which were 26% more likely to demonstrate aggression toward strangers. This large, comprehensive study of the relationships between gonadectomy and aggressive behavior in dogs demonstrates that when the many factors affecting aggressive behavior are considered, there is no evidence that gonadectomy at any age alters aggressive behavior toward familiar people or dogs, and there is only a minimal increase in aggression toward strangers. Given the increasing evidence of significant negative health effects of gonadectomy, there is an urgent need to systematically examine other means of preventing unwanted procreation, such as vasectomy and hysterectomy.

## Introduction

Aggressive behavior of dogs toward humans is a major public health hazard with significant animal welfare implications (1). Dog bites account for hundreds of thousands of emergency hospital visits annually, and children are particularly at risk (1–3). Dog–dog aggression is a widespread community concern (4), and it further increases the number of dog–human bites since many human bites occur when people try to separate fighting dogs (5). Aggression is a common reason for euthanasia of adult dogs (6).

Aggressive behavior of dogs can be broadly classified as directed toward people known to the dog (familiars), toward strangers, or toward other dogs. Canine aggression is manifested in a constellation of behaviors that can range from resource guarding to threatening and to attempted or successful bites, and there are many factors that modify canine aggression, including both environmental and genetic factors (7–10). The Canine Behavioral Assessment and Research Questionnaire (C-BARQ) is a well-validated series of questions to assess behavior, including aggressive behavior directed at familiars, strangers, and other dogs (11, 12).

Gonadectomy is widely used as a method to treat and prevent behavior problems, including problems related to aggression, in male and female dogs. At minimum, gonadectomy is advocated as a way to make dogs better-behaved companions. Major veterinary organizations, animal welfare groups, and shelter/rescue organizations promote these ideas (13–15). Behavior problems, including aggression, are the most common reason that owners relinquish adult dogs to shelters and rescue groups (6, 16–18). Relatively few published studies have specifically examined the effects of gonadectomy on aggressive behavior in domestic dogs, and the few studies that have provide conflicting information.

A number of studies have reported that gonadectomy neither prevents nor reduces dogs’ aggressive behavior. The first study to focus specifically on the effects of gonadectomy on canine aggression demonstrated that the frequency and intensity of aggressive behavior (competition for access to bones and to females in estrus) in prepubertally castrated males was not significantly different from that of intact littermate male controls (19). Borchelt conducted a retrospective study of 245 aggression-related behavior problems from data collected during private in-home consultations (20). While he noted that the number of intact and gonadectomized aggressive dog cases made it appear that intact males were more aggressive than neutered males and that spayed females were more aggressive than intact females, there was no significant difference between the ratio of intact and gonadectomized dogs and the number of aggression and nonaggression cases. O’Farrell and Peachy performed a prospective, controlled study in which a structured 40-question behavioral survey was administered to owners of 150 bitches at the time of spaying and again 6 months later and also twice 6 months apart to owners of 150 breed and age-matched intact bitches (21). The results showed that bitches younger than 1 year of age that had already displayed dominance aggression to family members had a 50:50 chance of increased aggressive behavior after ovariohysterectomy, whereas if left intact, they had a 6:1 chance of decreased aggressive behavior. These authors concluded that ovariohysterectomy should not be undertaken as a treatment for behavior problems and that ovariohysterectomy was associated with an increased risk of aggressive behavior.

Podberscek and Serpell studied the aggressive behavior of English Cocker Spaniels (22). Their analysis of 1,109 dogs initially found that gonadectomized males and females were more aggressive than intact dogs in several situations, including toward owners or members of the family, toward children in the household, and when reached for or handled. However, when they excluded dogs that were gonadectomized because they were aggressive, the differences in aggression between gonadectomized and intact dogs were no longer statistically significant. Another study also showed no discernable pattern for changes in aggression in 124 male dogs after gonadectomy (23). Guy et al. examined the demographic aspects of aggressive behavior in 3,226 dogs (24). The results showed that for dogs at least 1 year of age, neutered males had the highest odds of having bitten a member of the household, followed by spayed females and intact males. Similar trends were found for growling and resource guarding, which were considered to be signs of aggression. Spain et al. studied 1,842 male and female dogs gonadectomized and adopted between the ages of 6 and 12 months using a structured questionnaire sent 2–3 years later (25). During that period, aggression toward family members and strangers increased in males that were gonadectomized before 5.5 months. Reisner et al. investigated owner-directed aggression in a retrospective study of 1,053 adult English Springer Spaniels and found that aggressive behavior was significantly more likely to occur in gonadectomized dogs of both sexes than in intact dogs (26). Gonadectomized dogs of both sexes were significantly more likely than intact dogs to have bitten a person. Kim et al. conducted a prospective study of the effects of ovariohysterectomy on aggressive behavior in 14 German Shepherd dogs—7 were gonadectomized at the age of 5–10 months and 7 littermate controls remained intact (27). Ovariohysterectomized dogs demonstrated significantly more barking, growling, lunging, snapping, widened eyes, and lip lifting or curling in response to the approach of an unfamiliar human and dog. More recently, Garde et al. conducted a prospective study examining the behavioral effects of chemical (*n* = 30) and surgical castration (*n* = 36) on free-roaming dogs in Chile (28). The results demonstrated no reduction in aggressive behavior after surgical castration and a significant increase in dog-directed aggressive behavior after chemical castration.

By contrast, other studies seem to suggest that gonadectomy prevents or improves the aggressive behavior of dogs. In a prospective study, owners of 42 male dogs with at least one problem behavior including aggression toward other dogs chose elective gonadectomy, hoping that the procedure would decrease the dogs’ unwanted behaviors (29). Owners reported that gonadectomized dogs had a 62% decrease in aggressiveness toward other dogs but that territorial and fear-induced aggressive behaviors remained unaltered. The significance of these data could not be assessed because the study failed to include a control group of intact dogs with behavior problems for comparison. Further, owner’s responses were collected using an unstructured telephone interview an average of 27 months after surgery in which they were asked whether the dogs’ undesirable behavior had been altered by gonadectomy. Despite the limitations of the study, the authors concluded that the data were sufficient to justify gonadectomy as a therapeutic approach to solving undesirable behavior problems. Neilson and colleagues interviewed owners of 57 male dogs with behavior problems that were gonadectomized between 2 and 7 years of age for observed changes in behavior (30). There was no control group of intact dogs with behavior problems. Despite the fact that the statistical analysis demonstrated no significant results for any behavior examined, the authors concluded that castration had an effect on all behavior problems related to aggression, except for aggressive behavior toward strangers. The authors further recommended that veterinarians inform clients that 25% of male dogs should show a 50–90% improvement in aggressive behavior after castration.

The above studies represent some of the largest that have examined the effects of gonadectomy on aggressive behavior. It is worth noting that every prospective, controlled study that examined the effects of gonadectomy on the aggressive behavior of dogs demonstrated either no change in aggressive behavior or an increase in aggressive behavior after gonadectomy.

There has been increased interest in the effects of gonadectomy on the behavioral health of dogs in recent years. More groups are questioning the recommendation of gonadectomy as a routine procedure recommended across all pet dogs. The Board of Directors of the Society for Theriogenology and the American College of Theriogenology (2013) recommend that gonadectomy be decided on a case-by-case basis (31).

To date, there have been no published studies that used large numbers of dogs (i.e., several thousand) specifically to examine the effects of gonadectomy using operationally defined categories of aggressive behaviors, such as those described by the C-BARQ. There are also no studies that have examined potential differential effects of gonadectomy on canine behavior across age groups: puppies, juveniles, adolescents, and adults. The aim of the present study was to determine whether aggressive behavior toward familiar people, toward strangers, and toward other dogs was significantly different in dogs gonadectomized at different ages vs. intact dogs.

## Materials and Methods

### Study Population

The C-BARQ is a standardized evaluation tool used to assess dog behavior developed by researchers at the Center for the Interaction of Animals and Society at the University of Pennsylvania. The tool is available online (https://vetapps.vet.upenn.edu/cbarq/), and its presence and usefulness is widely advertised to veterinarians, behavioral consultants, researchers, shelters, breeders, dog owners and working dog organizations by a variety of means including the Internet and through word of mouth. The questionnaire has been demonstrated to be valid and reliable in assessing behavior and temperament traits in dogs (12, 32, 33) and has been used in a number of studies to analyze a variety of canine behaviors (34–36). It has been shown to be a reliable tool to assist in the selection of dogs for guide and service work, providing useful information with regard to training and breeding (35). The C-BARQ’s accuracy as a predictive behavioral tool provides an opportunity to draw upon a very large sample of dogs whose owners have answered a number of behavioral questions. This may have the effect of reducing bias compared with answering questions specifically pertaining to the effects of gonadectomy on aggressive behavior. There are 26 questions that address aggression in the C-BARQ (Table 1). Eight questions relate to aggression toward familiars, 10 to aggression toward strangers, and 8 to aggression toward other dogs.

**Table 1:** Relevant C-BARQ questions.

**Signalment questions** Dog’s name Dog’s age Breed Gender Approximate weight Where did you acquire this dog? *Possible answers (menu-driven)*: Bred him/her myself From a breeder From a shelter or a rescue group From a neighbor, a friend, or a relative Bought from a pet store Adopted him/her as stray Other At what age did you acquire this dog? Has this dog been spayed or neutered? If yes, at what age was this dog neutered? What was the primary reason for neutering this dog? *Possible answers (menu-driven)*: Birth control Required by a breeder, a shelter, or a rescue group To correct or reduce an existing behavior problem To correct or reduce an existing health problem To prevent future behavior problems To prevent future health problems Recommended by a veterinarian None of the above/don’t know Not applicable Is this dog currently suffering from any significant health problems? If yes, please describe briefly. Are you currently experiencing any problems with this dog’s behavior or temperament? Is (or was) this dog employed in any of the following activities or roles? *Possible answers (menu-driven)*: Breeding/showing Field trials/hunting Other sports (agility, racing, sledding, etc.) Working roles (search and rescue, service dog, sheepdog, etc.) None of the above Is this the first dog you have ever owned? If no, how many have you owned before Did you grow up with dogs as a child (0–16 years)? Are there other dogs in your household? If yes, are these other dogs *Possible answers (menu-driven)*: Older Younger Same age Older and younger Older and same Younger and same Older, younger, and same
**Questions relating to aggression**
Some dogs display aggressive behavior from time to time. Typical signs of moderate aggression in dogs include barking, growling, and baring teeth. More serious aggression generally includes snapping, lunging, biting, or attempting to bite.
By circling or underlining a number on the following 5-point scales (0 = No aggression, 4 = Serious aggression), please indicate your own dog’s recent tendency to display aggressive behavior in each of the following contexts: When verbally corrected or punished (scolded, shouted at, etc.) by you or a household member. (F)^a^ When approached directly by an unfamiliar *adult* while being walked/exercised on a leash. (S) When approached directly by an unfamiliar child while being walked/exercised on a leash. (S) Toward unfamiliar persons approaching the dog while s/he is in your car (e.g., at the gas station). (S) When toys, bones, or other objects are taken away by a household member. (F) When bathed or groomed by a household member. (F) When an unfamiliar person approaches you or another member of your family at home. (S) When unfamiliar persons approach you or another member of your family away from your home. (S) When approached directly by a household member while s/he (the dog) is eating. (F) When mailmen or other delivery workers approach your home. (S) When his/her food is taken away by a household member. (F) When strangers walk past your home while your dog is outside or in the yard. (S) When an unfamiliar person tries to touch or pet the dog. (S) When joggers, cyclists, rollerbladers, or skateboarders pass your home while your dog is outside or in the yard. (S) When approached directly by an unfamiliar male dog while being walked/exercised on a leash. (D) When approached directly by an unfamiliar female dog while being walked/exercised on a leash. (D) When stared at directly by a member of the household. (F) Toward unfamiliar dogs visiting your home. (D) Toward unfamiliar persons visiting your home. (S) When barked, growled, or lunged at by another (unfamiliar) dog. (D) When stepped over by a member of the household. (F) When you or a household member retrieves food or objects stolen by the dog. (F) Toward another (familiar) dog in your household (leave blank if no other dogs). (D) When approached at a favorite resting/sleeping place by another (familiar) household dog (leave blank if no other dogs). (D) When approached while eating by another (familiar) household dog (leave blank if no other dogs). (D) When approached while playing with/chewing a favorite toy, bone, object, etc., by another (familiar) household dog (leave blank if no other dogs). (D)

*^a^Letters in parentheses after the questions indicate whether the question addresses aggression toward familiar humans (F), toward strangers (S), or toward other dogs (D)*.

The owners of 15,370 dogs completed the C-BARQ questionnaire, and their answers represent the original data set for this study, which includes all surveys filled out between 2004 and June 1, 2011. Associations between gonadectomy and aggressive behavior were considered in several separate analyses. In each analysis, the outcome of interest was measured for three different types of aggressive behavior: aggressive behavior toward familiar people, aggressive behavior toward strangers, and aggressive behavior toward other dogs. In addition, the main independent variable of interest was evaluated in two different forms: dichotomous (gonadectomy status: no, yes) and polytomous [age at gonadectomy: 6 weeks to 6 months (puppy), 7–12 months (juvenile), 13–18 months (adolescent), more than 18 months (adult), or not gonadectomized]. For inclusion in the analyses presented, all survey questions relative to each type of aggression had to have been completed by the participant. These inclusion requirements were introduced to reduce possible bias in the conclusions.

Dogs were excluded from the study for the following reasons:

1. Any dog with incomplete data on any of the following 16 considered independent variables (is gonadectomized, the age at gonadectomy, why the dog was gonadectomized, the dog’s sex, where the dog was acquired, the dog’s health problems, concerns about behavior/temperament, the dog’s activity/role, first-owned dog, time owned, owned the dog as a child, other dogs in a household, the dog’s weight, the age the dog was acquired, and the age at evaluation) was removed from the data set (*n* = 857).
  Reason: these data include essential independent variables for the statistical analysis.
2. Any dog that was gonadectomized to correct a behavior problem was removed from the data set (*n* = 393).
  Reason: gonadectomy is frequently recommended by veterinarians to improve behavior, particularly aggressive behavior. By excluding dogs that had been gonadectomized to correct a behavior problem, we eliminated a potential confounding factor that would skew the data and might incorrectly suggest an association between gonadectomy and aggressive behavior, making it appear that neutered dogs were more aggressive.
3. Any dog reported to have been gonadectomized before 6 weeks was removed from the data set (*n* = 221).
  Reason: these dogs were excluded because very few dogs are gonadectomized at younger than 6 weeks, and publications discussing pediatric gonadectomy have reported studying only puppies 6 weeks of age or older. In addition, we had reason to suspect that these data represented inaccurate answers by the owners, because the majority stated that the dogs were gonadectomized at less than 3 weeks of age.
4. Any dog for which there were missing values for an outcome variable (aggression toward familiar people, aggression toward strangers, or aggression toward other dogs) was removed from that specific analysis. In cases in which specific questions were left unanswered because they were not applicable (e.g., a multiple-dog household question in a single-dog household), those data were left out of the analyses (*n* = 112).

Reason: these data include essential dependent variables for the statistical analysis.

### Variable Assessment

#### Independent Variables

The main independent variable of interest for this investigation was gonadectomy. This was examined in two separate ways: (a) gonadectomy status (no/yes) and (b) the age at gonadectomy (6 weeks to 6 months, 7–12 months, 13–18 months, older than 18 months, and not gonadectomized). These age groups correlate with stages of development: puppies, juveniles, adolescents, and adults. Animals listed as gonadectomized before 6 weeks were excluded as noted above.

Other independent variables that might have influenced the results of gonadectomy that were considered in further analyses were either categorical or continuous. The categorical variables were as follows: why gonadectomized, the dog’s sex, where the dog was acquired, the dog’s health problems, the dog’s activity/role, first dog-owned status, total number of dogs owned, dogs owned as a child, and other dogs in a household. The continuous variables were the dog’s weight, the age at gonadectomy, weeks since gonadectomy, the age the dog was acquired, and the age at evaluation.

#### Outcome Variable

Canine Behavioral Assessment Research Questionnaire data were used to define the categories for the outcome variable of interest: no aggression, mild aggression, moderate aggression, and severe aggression. Each of the types of aggression (i.e., toward familiar people, toward strangers, and toward other dogs) was evaluated on the basis of a set of questions relevant to that type. Possible scores ranged from 0 to 4. Answers for each aggression type were coded as follows: any response with at least one score of 3 or 4 was categorized as severe. Any response with at least one score of 2 was categorized as moderate unless it had already been categorized as severe. Any response with at least one score of 1 was categorized as mild unless it had already been categorized as moderate or severe. Finally, any response with scores of 0 for all questions was categorized as none. Further analyses were conducted to consider the effect of multiple variables; in these analyses, the moderate and severe categories were combined and the mild category was dropped, leaving two categories: moderate/severe and none. The purpose of this analysis was to perform a binomial comparison of the effects of gonadectomy on dogs with absolutely no evidence of aggression and those with significant levels of aggression.

### Statistical Analysis

Each independent variable was summarized within the level of aggression using means and standard deviations for the variables measured on a continuous scale. We tested for simple two-variable associations between gonadectomy/the age at gonadectomy and aggression using the χ^2^-test for categorical variables and the analysis of variance for continuous variables.

The analyses to consider and/or adjust for multiple variables used the outcome variables that were treated as binary (none vs. moderate/severe) for each type. For these analyses, we used a multivariable logistic regression model, which yielded adjusted odds ratios (ORs) as the measure of association for each independent variable considered with the outcome. Further model considerations included an adjustment for the repeated measures for observations from dogs within the same breed *via* the Huber/White sandwich estimator, yielding robust standard errors for statistical inference. Finally, regression models (ORs and associated *p*-values) presented include full models (i.e., all independent variables included in the model) and final or restricted models (i.e., independent variables restricted to those stated in the tables). The method of variable reduction used for these analyses was simple backward elimination from the full model based on *p* value (*p* < 0.10). In the modeling process to determine the final models, gonadectomy status and the age at gonadectomy, respectively, were held fixed in each of their respective models (i.e., not allowed to be eliminated). Analyses conducted using gonadectomy status did not include the variable age at gonadectomy and *vice versa*. Estimates for the effect of gonadectomy were calculated using six separate regression models—gonadectomy status and covariates with each of the three aggression outcomes and the age at gonadectomy and covariates with each of the three aggression outcomes. For statistical inference, *p* < 0.05 was considered to be significant, and all analyses were completed using Stata 13 statistical software (StataCorp, College Station, TX, USA).

## Results

Surveys of dog owners were conducted for 15,370 dogs. The final regression analyses were performed on 13,795 (89.7%) dogs for aggressive behavior toward familiar people, 13,498 (87.8%) dogs for aggressive behavior toward strangers, and 13,237 (86.1%) dogs for aggressive behavior toward other dogs.

Table 2 provides descriptive statistics for aggressive behavior toward familiar people according to each independent variable. The rightmost column of Table 2 lists the *p*-values as measures of the strength of the association between each independent variable listed and aggression toward familiars. In this simple (unadjusted) analysis, the dog’s age at gonadectomy had a statistically significant relationship with aggressive behavior toward familiar people (*p* < 0.001). Given the very large sample size for this analysis, almost all independent variables demonstrated a strong, statistically significant relationship with the outcome; the exceptions were the dog’s health problems, the dog’s age when acquired, and the dog’s age at evaluation. Tables 3 and 4 have the same format as Table 2. Table 3 presents the results for aggression toward strangers, and Table 4 presents the results for aggression toward other dogs. The age at gonadectomy was found to have strong, unadjusted, statistically significant relationships with increased aggressive behavior toward strangers (*p* < 0.001) and aggressive behavior toward other dogs (*p* = 0.001). Similarly, in Tables 3 and 4, most independent variables were shown to be significant, except the dog’s own health problems and the owner’s dog ownership as a child.

**Table 2:** Descriptive statistics for aggressive behavior toward familiar people according to exposure variables among 13,795 respondents.

Independent variable	Aggressive behavior severity score				*p*
	None (*n* = 9,717)	Mild (*n* = 2,368)	Moderate (*n* = 901)	Severe (*n* = 809)	
**Categorical variables, %**					
Is gonadectomized					<0.001
No	29.3	23.2	25.0	23.1	
Yes	70.7	76.8	75.0	76.9	
Age at gonadectomy categories					<0.001
Not gonadectomized	29.3	23.2	25.0	23.1	
6 weeks to 6 months	31.8	39.6	40.5	43.5	
>6–12 months	18.9	19.2	18.1	19.5	
>12–18 months	4.8	5.2	5.1	3.1	
>18 months	15.3	12.8	11.3	10.8	
Why gonadectomized					<0.001
Birth control	27.1	28.0	27.0	27.7	
Correct health problem	1.5	1.2	1.0	1.6	
Prevent health problems	7.0	6.6	5.3	4.1	
Prevent behavior problems	2.6	4.2	3.7	5.6	
Veterinarian recommended	3.7	6.0	7.4	9.4	
Required by a breeder	22.4	23.9	22.4	22.4	
Unknown	5.4	5.1	6.6	3.8	
N/A	30.5	25.0	26.6	25.5	
Dog’s sex					<0.001
Female	52.3	45.2	39.7	39.1	
Male	47.7	54.8	60.3	60.9	
Where the dog was acquired					<0.001
Breeder	48.6	44.1	38.5	37.7	
Stray	4.2	4.9	5.6	5.2	
Shelter	27.5	30.1	30.1	30.4	
Friend or relative	6.1	8.9	11.9	10.8	
Pet store	2.3	4.2	6.0	8.2	
Bred by owner	6.3	3.1	3.1	2.0	
Other	5.0	4.8	4.9	5.8	
Dog health problems					0.106
No	85.9	84.8	84.7	83.3	
Yes	14.1	15.2	15.3	16.7	
Dog’s activity/role					<0.001
None	59.6	71.0	78.3	84.4	
Working roles	6.3	3.8	2.9	2.0	
Field trials/hunting	2.2	1.8	0.9	1.7	
Breeding/showing	15.3	8.4	7.3	4.8	
Other sports	16.5	15.1	10.7	7.1	
First-owned dog					<0.001
No	86.9	80.7	78.3	77.6	
Yes	13.1	19.3	21.8	22.4	
Owned amount					<0.001
1 or 2	25.8	29.4	26.8	28.2	
3–5	30.0	28.8	28.9	31.9	
6–10	16.4	12.7	12.7	10.4	
>10	14.7	9.8	10.0	7.2	
N/A	13.1	19.3	21.8	22.4	
Owned dog as a child					0.046
No	18.5	19.7	20.8	21.6	
Yes	81.5	80.3	79.3	78.4	
Other dogs in a household					<0.001
No	28.6	37.5	40.2	44.4	
Yes	71.4	62.5	59.8	55.6	
**Continuous variables, mean (SD)**					
Dog’s weight	54.7 (31.2)	49.6 (30.1)	45.5 (31.7)	42.7 (31.7)	<0.001
Age at gonadectomy	47.1 (76.0)	43.0 (68.0)	41.3 (70.11)	40.1 (67.7)	0.002
Weeks since gonadectomy	179.0 (154.8)	185.2 (160.8)	193.2 (173.2)	194.3 (174.1)	0.003
Age the dog was acquired	40.8 (70.2)	43.5 (73.0)	41.9 (72.3)	45.3 (75.9)	0.166
Age at evaluation	226.1 (168.6)	228.1 (168.2)	234.5 (180.8)	234.5 (182.4)	0.316

*p-values were obtained using χ^2^-test for categorical variables and analysis of variance for continuous variables*.

**Table 3:** Descriptive statistics for aggressive behavior toward strangers according to exposure variables among 13,498 respondents.

Independent variable	Aggressive behavior severity score				*p*
	None (*n* = 3,392)	Mild (*n* = 4,519)	Moderate (*n* = 3,668)	Severe (*n* = 1,919)	
**Categorical variables, %**					
Is gonadectomized					<0.001
No	29.3	22.7	25.0	22.7	
Yes	70.7	77.3	75.0	77.3	
Age at gonadectomy categories					<0.001
Not gonadectomized	31.2	29.3	25.8	20.0	
6 weeks to 6 months	31.0	33.5	35.8	40.5	
>6–12 months	16.4	18.1	20.9	21.4	
>12–18 months	5.0	5.1	4.5	4.4	
>18 months	16.5	14.1	13.0	13.7	
Why gonadectomized					<0.001
Birth control	25.3	27.1	28.5	29.4	
Correct health problem	1.4	1.8	1.1	1.0	
Prevent health problems	6.7	7.0	6.9	5.6	
Prevent behavior problems	2.8	3.0	3.0	4.5	
Veterinarian recommended	3.8	3.9	5.4	6.6	
Required by a breeder	21.6	21.6	22.8	26.0	
Unknown	5.6	5.4	5.2	4.6	
N/A	32.8	30.3	27.0	22.3	
Dog’s sex					<0.001
Female	51.7	50.4	48.8	45.9	
Male	48.3	49.6	51.2	54.1	
Where the dog was acquired					<0.001
Breeder	50.0	51.3	45.0	35.4	
Stray	3.5	3.9	4.8	6.0	
Shelter	26.7	24.4	29.9	35.5	
Friend or relative	5.4	6.5	8.0	10.4	
Pet store	2.4	2.9	3.7	4.9	
Bred by owner	6.0	6.6	4.4	2.9	
Other	6.0	4.4	4.3	4.9	
Dog health problems					0.058
No	85.4	86.4	85.1	83.9	
Yes	14.6	13.6	14.9	16.2	
Dog’s activity/role					<0.001
None	58.3	58.6	67.9	77.9	
Working roles	6.9	5.6	4.9	3.7	
Field trials/hunting	2.2	2.7	1.5	1.6	
Breeding/showing	15.2	16.1	11.1	6.4	
Other sports	17.4	17.0	14.6	10.5	
First-owned dog					<0.001
No	85.6	86.7	83.2	81.4	
Yes	14.4	13.3	16.8	18.6	
Owned amount					<0.001
1 or 2	25.5	26.2	28.4	26.7	
3–5	29.3	30.3	29.3	32.4	
6–10	15.0	16.3	14.9	12.2	
>10	15.8	13.9	10.6	10.1	
N/A	14.4	13.3	16.8	18.6	
Owned dog as child					0.356
No	19.7	18.4	19.0	20.0	
Yes	80.3	81.6	81.1	80.0	
Other dogs in household					<0.001
No	29.8	29.2	33.3	37.4	
Yes	70.2	70.8	66.7	62.6	
**Continuous variables, mean (SD)**					
Dog’s weight	52.2 (29.6)	54.1 (31.1)	53.0 (33.0)	49.6 (31.4)	<0.001
Age at gonadectomy	50.8 (84.5)	44.9 (73.4)	42.5 (67.2)	45.3 (68.5)	<0.001
Weeks since gonadectomy	175.9 (163.2)	179.3 (155.9)	184.8 (153.0)	196.8 (160.6)	<0.001
Age the dog was acquired	47.9 (83.6)	36.1 (64.4)	38.8 (66.0)	46.3 (70.8)	<0.001
Age at evaluation	226.7 (180.5)	224.2 (168.0)	227.2 (163.1)	242.1 (166.7)	0.001

*p-values were obtained using χ^2^-test for categorical variables and analysis of variance for continuous variables*.

**Table 4:** Descriptive statistics for aggressive behavior toward other dogs according to categorical exposure variables among 13,237 respondents.

Independent variable	Aggressive behavior severity score				*p*
	None (*n* = 3,290)	Mild (*n* = 3,367)	Moderate (*n* = 3,328)	Severe (*n* = 3,252)	
**Categorical variables, %**					
Is gonadectomized					<0.001
No	30.8	31.9	27.1	20.9	
Yes	69.2	68.1	72.9	79.1	
Age at gonadectomy categories					<0.001
Not gonadectomized	30.8	31.9	27.1	20.9	
6 weeks to 6 months	33.7	32.6	33.0	37.5	
>6–12 months	18.0	17.8	18.9	21.1	
>12–18 months	4.9	4.2	5.1	5.3	
>18 months	12.7	13.5	16.0	15.3	
Why gonadectomized					<0.001
Birth control	26.4	24.6	28.5	29.5	
Correct health problem	1.0	2.0	1.5	1.2	
Prevent health problems	7.7	6.7	5.9	6.4	
Prevent behavior problems	2.5	2.9	3.1	3.9	
Veterinarian recommended	4.2	4.2	4.3	5.6	
Required by a breeder	21.1	20.8	22.8	25.5	
Unknown	5.0	5.6	5.6	5.4	
N/A	32.1	33.3	28.4	22.5	
Dog’s sex					0.008
Female	52.1	49.5	49.0	48.1	
Male	47.9	50.6	51.0	51.9	
Where the dog was acquired					<0.001
Breeder	53.1	51.1	47.2	36.8	
Stray	3.0	3.4	4.8	6.4	
Shelter	24.3	24.4	28.5	34.9	
Friend or relative	5.6	6.3	7.2	9.0	
Pet store	2.4	3.1	3.3	3.8	
Bred by owner	5.7	6.8	5.1	3.7	
Other	6.0	5.0	3.9	5.4	
Dog health problems					0.148
No	86.3	85.9	85.0	84.5	
Yes	13.7	14.1	15.1	15.5	
Dog’s activity/role					<0.001
None	59.8	57.7	63.2	72.8	
Working roles	7.5	5.8	4.7	4.1	
Field trials/hunting	2.4	2.6	1.8	1.6	
Breeding/showing	15.1	16.7	12.6	8.6	
Other sports	15.3	17.3	17.7	12.9	
First-owned dog					<0.001
No	86.4	85.5	83.0	82.9	
Yes	13.6	14.6	17.0	17.1	
Owned amount					<0.001
1 or 2	25.7	24.6	28.1	28.1	
3–5	29.7	29.6	28.9	30.9	
6–10	15.9	16.3	14.3	13.5	
>10	15.1	14.9	11.8	10.4	
N/A	13.6	14.6	17.0	17.1	
Owned dog as a child					0.303
No	18.8	18.4	20.1	19.3	
Yes	81.2	81.7	79.9	80.7	
Other dogs in household					<0.001
No	31.6	30.3	32.2	33.0	
Yes	68.4	69.7	67.8	67.0	
**Continuous variables, mean (SD)**					
Dog’s weight	53.4 (31.6)	53.3 (32.0)	51.2 (30.7)	52.3 (30.5)	0.010
Age at gonadectomy	43.4 (74.2)	42.9 (71.8)	48.6 (77.8)	48.6 (73.4)	0.001
Weeks since gonadectomy	157.3 (159.4)	185.0 (157.6)	190.3 (153.2)	202.5 (158.9)	<0.001
Age the dog was acquired	39.7 (72.6)	36.7 (67.1)	42.1 (72.0)	46.7 (71.4)	<0.001
Age at evaluation	200.7 (176.7)	227.9 (170.4)	238.9 (164.3)	251.0 (165.4)	<0.001

*p-values were obtained using χ^2^-test for categorical variables and analysis of variance for continuous variables*.

Tables 5 and 6 present OR estimates from multiple variable logistic regression models for each of the three aggression outcome variables. The outcome variable for the logistic regression analysis was transformed into a dichotomous variable (i.e., dogs with no reported aggression and dogs with moderate or severe aggression). The reason for this was so that the model could identify any relationships between gonadectomy and/or the age of gonadectomy and moderate to severe aggression because this level of aggression would be more likely to result in injury and/or would require behavioral intervention.

**Table 5:** Odds ratios (OR) and associated *p*-values from logistic regression models of aggressive behavior (none vs. moderate/severe) adjusted for all exposure variables (full models).

Independent variable	Aggression toward familiar people	Aggression toward strangers	Aggression toward other dogs
	OR (*p*) (*n* = 11,427)	OR (*p*) (*n* = 8,979)	OR (*p*) (*n* = 9,870)
**Is gonadectomized**			
No	Referent	Referent	Referent
Yes	1.201 (0.466)	1.059 (0.801)	0.903 (0.662)
**Age at gonadectomy categories**			
Not gonadectomized	Referent	Referent	Referent
6 weeks to 6 months	1.241 (0.377)	1.031 (0.897)	0.909 (0.701)
>6–12 months	0.978 (0.932)	1.181 (0.484)	1.011 (0.961)
>12–18 months	1.049 (0.872)	0.928 (0.757)	1.079 (0.773)
>18 months	0.842 (0.568)	1.132 (0.672)	1.401 (0.242)

*The model presented here was adjusted for why gonadectomized, the dog’s sex, where the dog was acquired, the dog’s health problems, the dog’s activity/role, first-owned dog, owned amount, owned the dog as a child, other dogs in a household, the dog’s weight, weeks since gonadectomy, the age the dog was acquired, and the age at evaluation*.

**Table 6:** Odds ratios (OR) and associated *p*-values from logistic regression models of aggressive behavior (none vs. moderate/severe) adjusted for significant exposure variables *via* backward selection (restricted models).

Independent variable	Aggression toward familiar people	Aggression toward strangers	Aggression toward other dogs
	OR (95% CI)	OR (95% CI)	OR (95% CI)
	*p* (*n* = 11,427)	*p* (*n* = 8,979)	*p* (*n* = 9,870)
**Is gonadectomized**			
No	Referent^a^	Referent^b^	Referent^c^
Yes	1.15 (0.71, 1.85) 0.577	1.22 (1.04, 1.43) 0.014	0.89 (0.58, 1.39) 0.617
**Age at gonadectomy categories**			
No gonadectomy	Referent^d^	Referent^e^	Referent^f^
6 weeks to 6 months	1.24 (0.78, 1.98) 0.357	1.14 (0.96, 1.34) 0.130	0.91 (0.57, 1.46) 0.689
7–12 months	0.98 (0.61, 1.59) 0.933	1.26 (1.09, 1.45) 0.002	1.01 (0.68, 1.51) 0.965
13–18 months	1.05 (0.61, 1.82) 0.861	0.95 (0.78, 1.15) 0.586	1.08 (0.67, 1.74) 0.752
>18 months	0.84 (0.52, 1.34) 0.463	0.98 (0.80, 1.12) 0.850	1.44 (0.92, 2.27) 0.115

*^a^This model was adjusted for why gonadectomized, the dog’s sex, where the dog was acquired, the dog’s activity/role, owned amount, other dogs in a household, the dog’s weight, and weeks since gonadectomy*.

*^b^This model was adjusted for the dog’s sex, where the dog was acquired, the dog’s activity/role, owned amount, weeks since gonadectomy, the age the dog was acquired, and the age at evaluation*.

*^c^This model was adjusted for why gonadectomized, the dog’s sex, where the dog was acquired, the dog’s activity/role, owned amount, the age the dog was acquired, and the age at evaluation*.

*^d^This model was adjusted for why gonadectomized, the dog’s sex, where the dog was acquired, the dog’s activity/role, owned amount, other dogs in a household, the dog’s weight, and the age at evaluation*.

*^e^This model was adjusted for the dog’s sex, where the dog was acquired, the dog’s activity/role, owned amount, the age the dog was acquired, and the age at evaluation*.

*^f^This model was adjusted for why gonadectomized, the dog’s sex, where the dog was acquired, the dog’s activity/role, owned amount, weeks since gonadectomy, and the age the dog was acquired*.

Table 5 presents the results from regression models that included all independent variables considered as listed in Tables 2–4, referred to as full models. For aggression toward familiar people, gonadectomy status was shown to have no effect on the dog’s aggressive behavior (OR near 1.20) and was given no statistical significance (*p* = 0.466). In the regression model of aggression toward familiars using the age at gonadectomy (instead of gonadectomy status), the age at gonadectomy also showed no statistical significance with the outcome variable.

For aggression toward strangers, gonadectomy status also showed no protective effect against aggression (OR = 1.06) and was not significant (*p* = 0.801). In addition, the age at which the dog was gonadectomized was not statistically significant in any age category and thus was interpreted as showing no effect.

Finally, for aggression toward other dogs, the OR for gonadectomy was also not significant (OR = 0.90, *p* = 0.104), and the age at gonadectomy showed no association with aggression in any category of age.

Table 5 demonstrates that, for the fully adjusted regression models, the ORs estimated from the logistic model of aggressive behavior do not identify a change in the odds (risk) of having moderate or severe aggressive behavior as opposed to no aggressive behavior for any gonadectomy status.

The format of Table 6 is similar to that of Table 5, but the results shown are from multiple variable regression models that were restricted to significant independent variables *via* the simple backward variable elimination process. Neither gonadectomy nor the age at gonadectomy showed an association with aggression toward familiar people or other dogs. However, for aggression toward strangers, an association was found that demonstrated a low but significant (22%) increase in the odds of moderate or severe aggression among gonadectomized dogs compared with intact dogs (OR = 1.219, *p* = 0.014). This increase in aggression to strangers on the part of gonadectomized dogs was driven entirely by data for dogs gonadectomized between 7 and 12 months of age. Dogs in that age group were significantly more likely to demonstrate aggression toward strangers (OR = 1.259, *p* = 0.002).

## Discussion

This study is the largest to date to examine how gonadectomy, which was performed at different ages, affects the aggressive behavior of domestic dogs. Initial analysis identified independent variables that might influence aggressive behavior, and these factors were then used for multivariable analysis. Two logistic regression models were used. In the logistic regression model that was adjusted for all variables, there was no significant relationship between aggressive behavior toward familiar people, strangers or other dogs, and (a) gonadectomy status or (b) the dog’s age at gonadectomy. In the logistic regression model adjusted for significant exposure variables, there was just one age-related effect of gonadectomy; dogs that were gonadectomized between 7 and 12 months of age were significantly more likely to show aggression to strangers. Dogs gonadectomized at other ages did not show aggression to strangers. Further, dogs gonadectomized at any age did not show aggression to familiar people or other dogs.

It is difficult to explain why our analysis demonstrated a significant increase in aggression toward strangers in dogs gonadectomized between 7 and 12 months of age. It is possible that this is a type I error or false-positive finding related to the use of two different models. It is also possible that the experience of gonadectomy at this age creates a long-lasting fear response to strangers—it is difficult to know. Regardless, these data certainly do not suggest that intact dogs, compared with gonadectomized dogs, exhibit increased aggression to familiar people, strangers, or other dogs. They also do not support the view that gonadectomy will predictably reduce the dogs’ aggressive behavior. This lends further support to the idea that current assumptions about sex and the aggressive behavior of dogs might be predicated on anecdotal beliefs (e.g., that the aggressive behavior of dogs can be predicted by testosterone levels).

The literature also supports the view that multiple factors influence aggressive behavior, regardless of a dog’s reproductive status. For example, Perez-Guisado and Munoz-Serrano found owner-dependent factors to be more significant than dog-dependent factors in influencing the aggressive behavior of dogs (37). Their studies showed more aggressive behavior from dogs owned by first-time dog owners, dogs with less obedience training, and dogs acquired as a gift or to guard. McMillan et al. found more aggressive behavior toward familiar people, strangers, and other dogs in puppies acquired from pet stores than from noncommercial breeders (38). Guy et al. found yet other environmental factors correlated with aggression, including dogs in homes with one or more teenagers and dogs that had a history of skin disorders (39). Roll and Unshelm studied dog interactions and found differences between the owners of dog aggressors and those of dog victims regarding gender, profession, and the purpose for which the owner acquired the dog (40). They also found that obedience training and the owner’s attitude toward training were significant factors. Hsu and Sun found that dogs that had received physical punishment displayed higher levels of aggressive behavior (34). More owner-directed aggression was found (a) toward female owners than toward male owners and (b) when dogs were kept outside. In view of these findings, and the findings of the current study, it is essential to look beyond reproductive status to identify the causes and discover solutions for aggressive behavior exhibited by dogs.

A major variable that was purposely not included in the current study was differences in aggressive behavior between dog breeds (32, 41). Given that dogs have been bred for different tasks and display different breed-specific tendencies, a direction for future research would be to determine whether and how different breeds might respond differently to gonadectomy. Reproductive status has often been included in reports of dog-bite incidents and dog-bite fatalities. Overall and Love discussed the epidemiology of dog bites and referred to the reproductive status of biting dogs when examining the many factors that may contribute to dog bites and fatalities (42). In their analyses, intact male dogs were more likely to bite than neutered male dogs. However, they found many other risk factors that were also associated with the probability of a dog-bite incident. For example, unlicensed dogs were also more likely to bite, as were dogs left chained in the yard and dogs not current on their vaccinations. Reproductive status was viewed as one of many factors in a dog’s history that would determine the expression of that individual dog’s behavior. In their study of fatalities related to dog bites, Patronek et al. reported that dogs involved with dog-bite fatalities had seven factors in common that were more significant than breed (43). At least four of the following factors were present in more than 80% of the fatalities (in no particular order): the dog was kept isolated from a regular positive interaction with humans rather than being a family dog, the owner had a history of prior mismanagement of dogs, the owner had a history of abuse or neglect of dogs, the victim had little or no relationship with the dog, the victim had a compromised ability to interact appropriately with the dog, an able-bodied individual was not present to intervene during the attack, and the owner failed to neuter the dog. When all factors are considered, the data that intact male dogs are more likely to bite might have more to do with the attitudes and behavior of their owners than the possession of intact gonads.

Recently, a number of studies have presented new data with respect to the health risks of gonadectomy. While gonadectomized dogs experience a zero risk of ovarian or testicular cancer and a lowered risk of benign prostatic hyperplasia and anal gland adenocarcinomas, there is mounting evidence that gonadectomy significantly increases the risks of developing many different serious forms of cancer, including hemangiosarcoma, mast cell cancer, prostatic carcinoma, osteosarcoma, and lymphoma/lymphosarcoma, along with the development of these cancers at earlier ages (44–55). This increased risk of cancer may be related to the long-term effects of elevated blood levels of luteinizing hormone (LH) (56). LH binds to receptors on many tissues throughout the body; this reaction stimulates a number of cellular processes including cell division and nitric oxide release. In addition, gonadectomized dogs have increased risks of a number of orthopedic diseases, including cranial cruciate-ligament insufficiency, hip dysplasia and patellar luxation (44–46, 57–61), and also of numerous autoimmune diseases (62).

Some studies have shown that intact dogs (particularly males) are more prone to roaming behavior (29, 63), which can result in vehicular injury. A more recent study that examined the behaviors of chemically or surgically castrated free-roaming dogs in Chile using GPS collars indicated that the gonadectomized dogs had no change in the size of their home range, regardless of the method of castration (28). The numerous issues surrounding dog overpopulation are beyond the scope of this study. Nonetheless, the literature suggests a critical need to study the health effects of other means by which dogs can be prevented from producing offspring, including vasectomy in males and hysterectomy in females.

The results of this study and a thorough search of the literature demonstrate that no single factor is responsible for the aggressive behavior of dogs but instead that there are multiple environmental and genetic factors that contribute to aggressive behavior. Clearly, gonadectomy does not result in a predictable decrease in aggressive behavior across all male and female dogs, although it might be effective in some. This emphasizes the importance of individualized veterinary medicine, in which the decision whether or not to gonadectomize an individual dog is based on an assessment of many different environmental and genetic factors.

Further, a successful strategy to decrease aggressive behavior in populations of dogs will require a comprehensive approach to educating the public about the full responsibilities of dog ownership, which includes such factors as how to find and select an appropriate dog, a dog’s daily physical and mental requirements, the amount of time required for basic training, and the cost of basic health care. It is our hope that this study will inspire more research concerning the effects of gonadectomy on dog behavior, particularly studies using randomized, controlled trials to account for the effects of environmental factors. Perhaps even more optimistically, we hope that these findings might provide a place of convergence for people who share a common goal: to improve the quality of life of domestic dogs and their owners.

## Ethics Statements

Does the study presented in the manuscript involve human or animal subjects: yes. This study was carried out in accordance with the standards set by the Institutional Review Board (IRB) and the Institutional Animal Care and Use Committee (IACUC) at Hunter College of the City University of New York. Individuals completing C-BARQ give full consent for results to be used for research.

## Author Contributions

PF conceived the study. JS and DD collected the data. IM, PT, PF, and CZ analyzed the data. PF and CZ wrote the manuscript. All the authors contributed to the revision of the manuscript and approved the final version of the manuscript for submission.

## Conflict of Interest Statement

The authors declare that the research was conducted in the absence of any commercial or financial relationships that could be construed as a potential conflict of interest.
